# Integrated metabolome and transcriptome analyses reveal the role of *BoGSTF12* in anthocyanin accumulation in Chinese kale (*Brassica oleracea* var. *alboglabra*)

**DOI:** 10.1186/s12870-024-05016-5

**Published:** 2024-04-25

**Authors:** Kang Tang, Umer Karamat, Guihua Li, Juxian Guo, Shizheng Jiang, Mei Fu, Xian Yang

**Affiliations:** 1 College of Horticulture, South China Agricultural University, Guangzhou, 510642 China; 2 Guangdong Key Laboratory for New Technology Research of Vegetables, Vegetable Research Institute, Guangdong Academy of Agricultural Sciences, Guangzhou, 510642 China

**Keywords:** Metabolome, RNA-seq, Anthocyanins, qRT-PCR, *Brassica oleracea*

## Abstract

**Background:**

The vivid red, purple, and blue hues that are observed in a variety of plant fruits, flowers, and leaves are produced by anthocyanins, which are naturally occurring pigments produced by a series of biochemical processes occurring inside the plant cells. The purple-stalked Chinese kale, a popular vegetable that contains anthocyanins, has many health benefits but needs to be investigated further to identify the genes involved in the anthocyanin biosynthesis and translocation in this vegetable.

**Results:**

In this study, the purple- and green-stalked Chinese kale were examined using integrative transcriptome and metabolome analyses. The content of anthocyanins such as cyanidin-3-*O*-(6″-*O*-feruloyl) sophoroside-5-*O*-glucoside, cyanidin-3,5-*O*-diglucoside (cyanin), and cyanidin-3-*O*-(6″-*O*-*p*-hydroxybenzoyl) sophoroside-5-*O*-glucoside were considerably higher in purple-stalked Chinese kale than in its green-stalked relative. RNA-seq analysis indicated that 23 important anthocyanin biosynthesis genes, including 3 *PAL*, 2 *C4H*, 3 *4CL*, 3 *CHS*, 1 *CHI*, 1 *F3H*, 2 *FLS*, 2 *F3’H*, 1 *DFR*, 3 *ANS*, and 2 *UFGT*, along with the transcription factor *BoMYB114*, were significantly differentially expressed between the purple- and green-stalked varieties. Results of analyzing the expression levels of 11 genes involved in anthocyanin production using qRT-PCR further supported our findings. Association analysis between genes and metabolites revealed a strong correlation between *BoGSTF12* and anthocyanin. We overexpressed *BoGSTF12* in *Arabidopsis thaliana tt19*, an anthocyanin transport mutant, and this rescued the anthocyanin-loss phenotype in the stem and rosette leaves, indicating *BoGSTF12* encodes an anthocyanin transporter that affects the accumulation of anthocyanins.

**Conclusion:**

This work represents a key step forward in our understanding of the molecular processes underlying anthocyanin production in Chinese kale. Our comprehensive metabolomic and transcriptome analyses provide important insights into the regulatory system that controls anthocyanin production and transport, while providing a foundation for further research to elucidate the physiological importance of the metabolites found in this nutritionally significant vegetable.

**Supplementary Information:**

The online version contains supplementary material available at 10.1186/s12870-024-05016-5.

## Introduction

One of the most economically important crop families, Brassicaceae, contains 372 genera and 4,060 species, including *Brassica oleracea*, *B. napus*, *B. rapa*, and the model plant *Arabidopsis thaliana* [1]. Brassicaceae crops are consumed by people all over the world and are rich in many nutritionally beneficial compounds, including phenolics, vitamins, anthocyanins, glucosinolates, and carotenoids. Due to valuable concentrations of phenolic components, ascorbic acid, tocopherols, and carotenoids, these vegetables are a natural source of antioxidants that help protect the body from damage caused by reactive oxygen species [2–6].

Purple-stalked kale (*B. oleracea* var. *alboglabra*) is a variety of *B. oleracea*. Its color results from the presence of anthocyanins in the stalk epidermal cells [7]. Anthocyanins are water-soluble pigments that occur in a variety of plants and serve a wide range of biological purposes including coloration and stress protection. They give plants their characteristic red, pink, purple, or blue colors, and they also play important roles in resistance to biotic and abiotic stresses [8, 9]. Additionally, anthocyanins have potent antioxidant capabilities that are advantageous for human health [10]. A significant amount of research has been conducted to understand the anthocyanin biosynthesis pathway and its regulation. Key genes in the pathway include *phenylalanine ammonia lyase* (*PAL*), *chalcone acetylase* (*CHS*), *chalcone isomerase* (*CHI*), *flavanone 3-hydroxylase* (*F3H*), *flavonoid 3’-hydroxylase* (*F3’H*), *dihydroflavonol 4-reductase* (*DFR*), *anthocyanin synthase* (*ANS*), and *UDP-glucose: flavonoid 3-O-glucosyltransferase* (*UFGT*) [11, 12]. In addition, WRKY, basic helix-loop-helix protein (bHLH), MYB, WD40, and NAC transcription factors (TFs) have been identified and studied in higher plants as important in anthocyanin biosynthesis [13, 14]. Several different TFs control the production of anthocyanins in plants. Typically, MYB transcription factors that are involved in anthocyanin production belong to the subgroup MYB-bHLH-WD40 (MBW complex) [15]. Three different TF types make up this complex: WD40 repeat, bHLH, and MYB proteins [15]. Expression of the genes involved in anthocyanin biosynthesis is activated by the combined action of these transcription factors.

Additionally, environmental variables are closely linked to anthocyanin biosynthesis, metabolism, and storage [16], with temperature being a key external signal. In general, during low temperatures, genes involved in anthocyanin synthesis in plants are stimulated, resulting in an increase in anthocyanin content, whereas high temperatures can speed up anthocyanins degradation and result in the fading of plant color. Additionally, it has been demonstrated that some plants accumulate anthocyanins as a defense against adverse environmental conditions, such as cold temperatures [17–21].

Within the Brassicaceae family, genes related to anthocyanin synthesis and regulation have been identified mainly through mapping and transcriptome sequencing. In *B. rapa*, *BrMYB2* [22, 23], *BrMYBL2.1* [24], *BrMYB114* [25], *BrbHLH49* [26], and *BrEGL3.2* [27] are associated with the purple trait. In *B. juncea*, *BjTT8* controls the color of purple tumorous stem mustard [28], and *BjPl1* is related to its purple leaf color [29]. In *(A) thaliana*, the transcription factors MYB114, MYB113, and MYB118 are involved in controlling the production of anthocyanins [30]. A number of genes that control some of the leaf colors in *(B) oleracea* have been identified: it was shown that *DFR* and *Re* genes control the red-leaf characteristic [31–34], and *BoMYB2* regulates the production of the purple color in leaves of ornamental kale [35]. Although anthocyanins biosynthesis in *B. oleracea* is clear, anthocyanin accumulation is less understood.

In this study, using transcriptome and metabolome association analyses, we identified *BoGSTF12* gene. Biochemical, genetic and molecular studies showed that *BoGSTF12* was a transporter of anthocyanin. These findings will improve our understanding of the regulation of anthocyanin accumulation in Chinese kale and also provide useful gene resources for breeding.

## Materials and methods

### Plant materials and phenotypic analysis

Inbred lines of purple-stalked (HJJL, R) and green-stalked (ZSJL, G) Chinese kale (*B. oleracea* var. *alboglabra*) were used as the experimental materials in this study. The original seeds were provided by the Vegetable Research Institute, Guangdong Academy of Agricultural Sciences. Both varieties of Chinese kale were grown at the Baiyun experimental fields of the Vegetable Research Institute, Guangdong Academy of Agricultural Sciences, Guangzhou, China. The skin of the stalks was peeled off with a blade, then quickly stored in liquid nitrogen. Samples were collected at the same time for metabolome analysis, RNA sequencing (RNA-seq), and qRT-PCR validation. Three biological replicates were taken from each group of samples, each biological replicate consisting of a mixture of five plants.

### Metabolite identification

A vacuum freeze-dryer (Scientz-100 F) was used to dry skin. The freeze-dried samples were ground using zirconium beads for 1.5 min at 30 Hz in a mixer mill (MM 400, Retsch). Then, 100 mg of lyophilized powder was dissolved in 1.2 mL of 70% methanol in water, vortexed six times for 30 s each (once every 30 min), and then stored at 4 °C overnight. Before ultra-high performance liquid chromatography-mass spectrometry (UPLC-MS)/MS analysis, the extracts were filtered (SCAA-104, 0.22 μm pore size) after centrifugation at 12,000 rpm for 10 min.

An UPLC-ESI-MS/MS system (UPLC, SHIMADZU Nexera X2; MS, Applied Biosystems 4500 Q TRAP) was used to analyze the sample extracts. The following analytical conditions were used: an Agilent SB-C18 column, 1.8 μm, 2.1 mm×100 mm; Solvent A, sterile clean water with 0.1% formic acid, and Solvent B, acetonitrile with 0.1% formic acid, made up the mobile phase. The starting conditions were 95% A and 5% B, followed by a linear gradient to 5% A, 95% B in less than 9 min, with the composition of 5% A, 95% B maintained for 1 min. Then, within 10 min, a composition of 95% A and 5% B was used and maintained for 2 min. The chosen flow velocity was 0.35 mL/min, the injection volume was 4 µL, and the column oven was adjusted to 40 °C. An alternate connection was made between the effluent and the electrospray ionization (ESI)-triple quadrupole-linear ion trap (QTRAP)-MS.

QTRAP UPLC/MS/MS System was equipped with an ESI Turbo Ion-Spray interface, operating in positive and negative ion modes, and was managed by Analyst 1.6.3 software (AB Sciex); linear ion trap (LIT) and triple quadrupole (QQQ) scans were acquired. The following ESI source operation parameters were used: Turbo spray ion source; 550 °C source temperature; 5500 V (positive ion mode)/−4500 V (negative ion mode) ion spray voltage; 50, 60, and 25 psi, respectively, for the ion source gas I (GSI), gas II (GSII), and curtain gas (CUR); and high collision-activated dissociation (CAD). The instrument calibration and mass calibration were carried out using solutions of 10 and 100 mol/L polypropylene glycol in the QQQ and LIT modes, respectively. For multiple reaction monitoring (MRM) investigations, QQQ scans were recorded with the collision gas (nitrogen) set to medium. Further declustering potential (DP) and collision energy (CE) optimization was used to perform DP and CE for individual MRM transitions. According to the metabolites eluted during each interval, a particular set of MRM transitions were observed.

### RNA-seq analysis

Total RNA from stalk skin was isolated using an RNA Extraction Kit (Tiangen, Beijing, China). The Agilent Bioanalyzer 2100 system (Agilent Technologies, Palo Alto, CA, USA) was used to confirm the amount of RNA present. Using the purple- and green-stalked Chinese kale, six cDNA libraries (R1, R2, R3, G1, G2, and G3) were constructed. All samples were sequenced on the Illumina NovaSeq 6000 platform, PE150 model. The manufacturer’s instructions were followed in the construction and sequencing of the RNA-seq library, as previously described [36]. The adapter and low-quality sequences were removed from the raw readings. Clean reads were successfully mapped to the *Brassica* reference genome. Gene expression levels were calculated using the fragments per kilobases per million fragments (FPKM) technique. The following thresholds were used to identify DEGs: |log2(foldchange)| ≥ 1 and FDR < 0.01. Gene Ontology (GO) and KEGG enrichment analyses of DEGs were performed using the cluster Profiler R package (http://www.geneontology.org/) and the Kyoto Encyclopedia of Genes and Genomes (KEGG) http://www.genome.ad.jp/kegg/.

### qRT-PCR analysis

Total RNA was isolated from stalk skin using a Megan RNA Extraction Kit (Guangzhou Magen Biotechnology Co., Ltd.). Table S1 contains a list of the PCR primers used in this work. The control was the actin gene. Each qRT-PCR reaction contained 0.3 µL of the appropriate primers, 1 µL of template cDNA, 3.4 µL of ddH_2_O, and 5 µL of 2× ChamQ Universal SYBR qPCR Master Mix (Vazyme, Nanjing, China) in a total volume of 10 µL.

### Integrated metabolome and transcriptome analyses

Correlation coefficients were computed between the metabolome and transcriptome datasets. These coefficients were derived from the log2-fold changes of individual metabolites and transcripts, using the EXCEL program. Cytoscape version 2.8.2 was used to visualize the relationships between the metabolome and transcriptome.

### Statistical evaluation

The statistical analysis of variance was performed using GraphPad Prism8.0.lnk. The mean and standard deviation for three biological replicates of the data were displayed. The threshold point for significant differences was determined at *p* < 0.05.

### Overexpression of *BoGSTF12* in the *Arabidopsis tt19* mutant

Using gene-specific primers (Table S1), the target gene was cloned and then transferred into the pCAMBIA1301 binary vector. Using the freeze–thaw technique, the vectors generated were introduced into *Agrobacterium tumefaciens* strain GV3101 before being transformed into the *Arabidopsis tt19* mutant. The transgenic seeds were cultivated for 7 days on half-strength Murashige and Skoog plates with hygromycin before being planted. T3 homozygous lines were produced for future investigation. At various developmental stages, the phenotypic traits of the transgenic, mutant, and *Arabidopsis* wild-type (WT) plants were observed. To determine the anthocyanin contents, we used a method described in a previous study [37]. Samples were frozen in liquid nitrogen and were subsequently ground into a fine powder using a mortar and pestle under liquid nitrogen. After mixing the powder with 1% HCl in methanol, samples were incubated for 24 h at 4 °C. Anthocyanins were quantified by measuring absorbance at 530 and 657 nm.

### Phylogenetic analysis

The protein sequences of GSTs from several species were used to construct a phylogenetic tree by MEGA 5 using the neighbor-joining method with a bootstrap value of 1000. Sequences used for phylogenetic tree analysis are listed in Table S2.

## Results

### Phenotypic analysis and anthocyanin metabolite identification in purple- and green-stalked Chinese kale

**Fig. 1:**
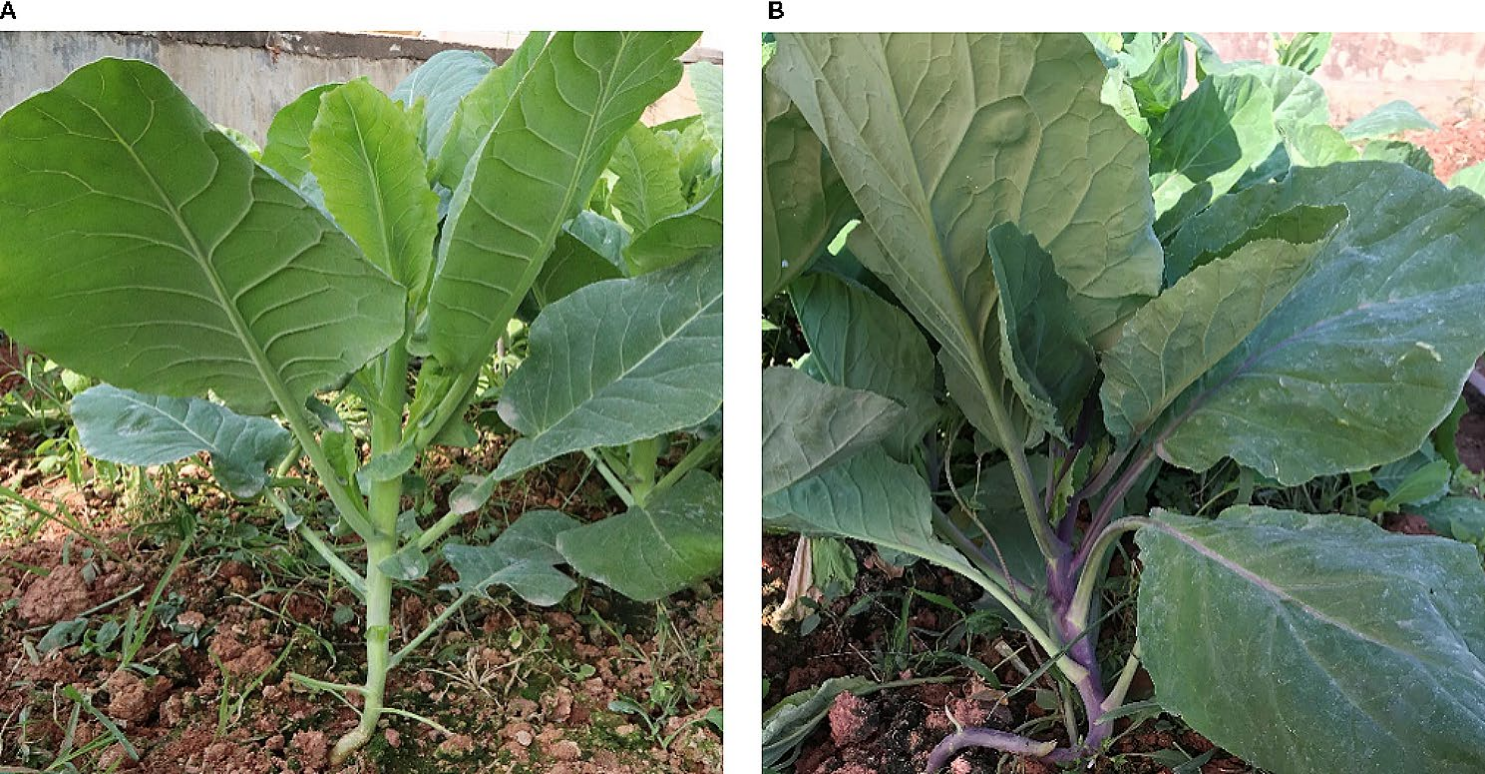
Comparison of two Chinese kale phenotypes. (**a**) Green-stalked Chinese kale and (**b**) purple-stalked Chinese kale. Photographs show seven -week- old plants grown in the field as described in Methods

The most striking difference between the two varieties was the coloration of their stalks. The purple-stalked Chinese kale displayed a rich and vibrant purple color, whereas the green-stalked variety exhibited a lush and vivid green color (Fig. 1). Detailed genetic and biochemical analysis of these phenotypic differences can offer valuable insights into the physiological bases of these traits and potential implications for breeding and culinary applications of Chinese kale varieties.

**Fig. 2:**
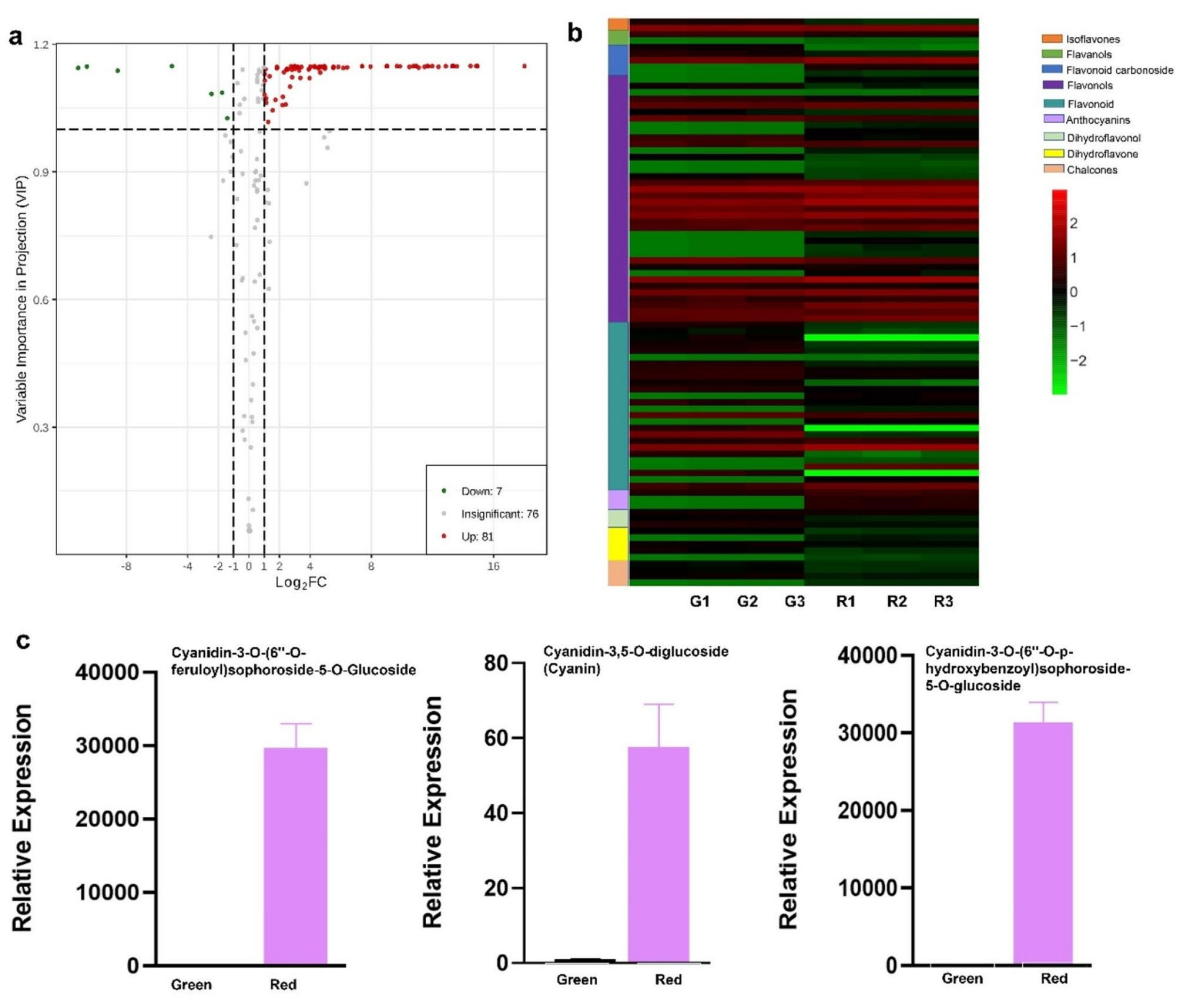
Differential levels of anthocyanin metabolites in purple- vs. green-stalked Chinese kale. (**a**) Volcano plot demonstrating the statistical significance of the differences in metabolite levels in the two Chinese kale varieties. (**b**) Heatmap comparing metabolite contents in purple and green-stalked Chinese kale. Colors represent differential expression levels after normalization. The left side of the heatmap shows the metabolite classes as identified in the color key to the right. G1–G3 and R1–R3 represent the different samples. (**c**) Anthocyanins of differential metabolites in two types of Chinese kale

The anthocyanin levels of the purple- vs. green-stalked Chinese kale differed noticeably. We performed a thorough metabolomic analysis to discover differences in the contents of anthocyanin-related metabolites between purple and green-stalked Chinese kale. A total of 88 differentially occurring metabolites were found, with 81 at higher levels and 7 at lower levels (Fig. 2a) in purple-stalked kale, which had more overall anthocyanin production than the green-stalked variety (Fig. 2b). The levels of anthocyanin-related compounds such as cyanidin-3-*O*-(6″-*O*-feruloyl) sophoroside-5-*O*-glucoside, cyanidin-3,5-*O*-diglucoside (cyanin), and cyanidin-3-*O*-(6″-*O*-*p*-hydroxybenzoyl) sophoroside-5-*O*-glucoside were significantly and prominently higher in the purple-stalked plants than in the green-stalked ones (Fig. 2c and Table S3).

### Transcriptome analysis

**Fig. 3:**
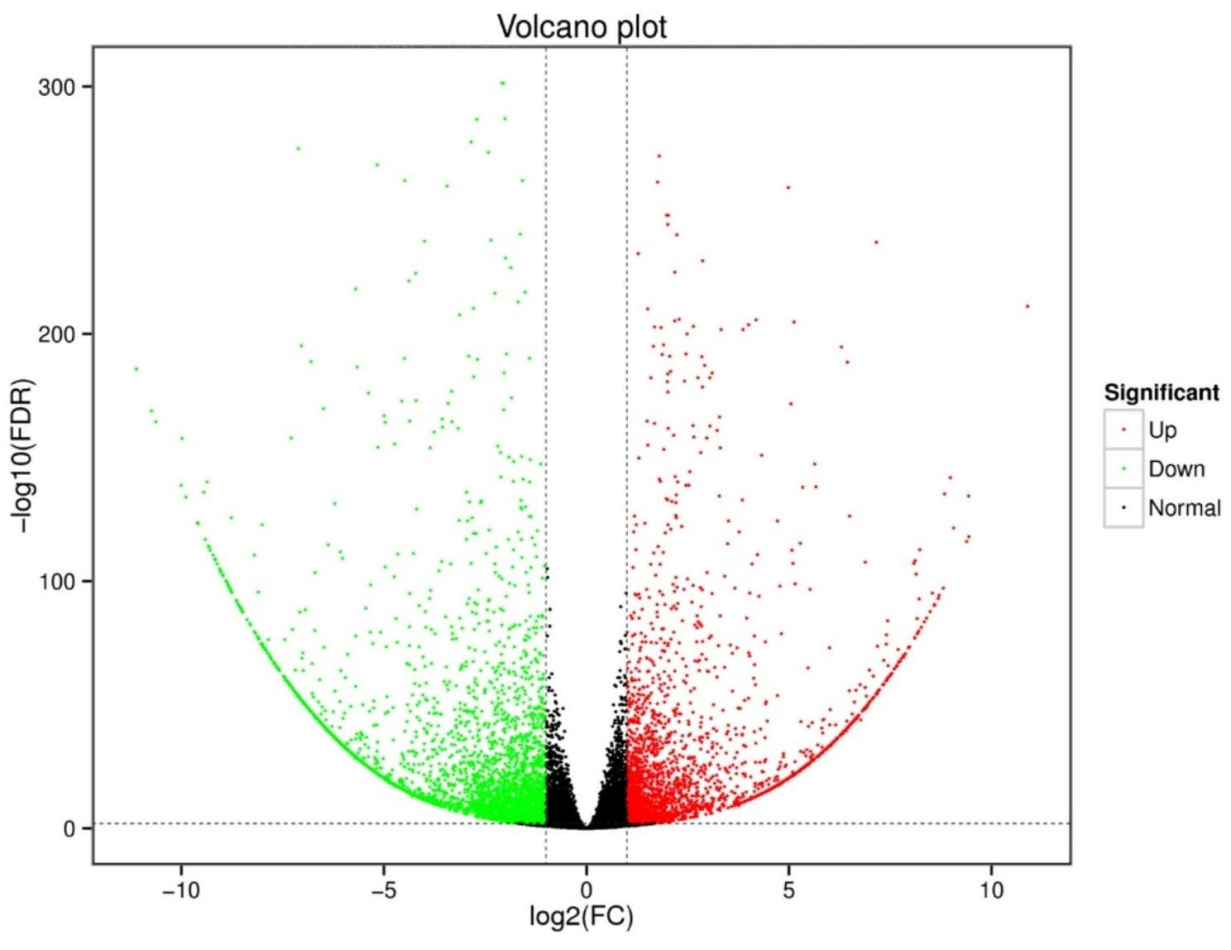
Volcano plot showing DEGs between purple- and green-stalked Chinese kale varieties

Skin tissues of the purple- and green-stalked Chinese kale stalks were used to construct cDNA libraries, and RNA-seq analysis was carried out to determine the molecular process behind the production of anthocyanins in the samples. More specifically, the clean data for each sample amounted to 5.78 Gb, for a total of 37.22 Gb, and the Q30 baseline percentage was 94.74% and above (Table S4). Clean reads for each sample were sequenced along with the designated reference genome, and the mapped reads were between 91.19% and 92.02% (Table S5). Variable splicing prediction analysis, gene structure optimization analysis, and new gene discovery were done based on the comparison results, and 3,878 new genes were found. Of these, 2,404 genes were found to have functional annotations. In our differential gene expression analysis, we found a total of 5,826 DEGs, of which 2,408 were upregulated and 3,418 were downregulated in the purple-stalked variety relative to the green-stalked variety (Fig. 3, Table S6).

### Functional annotation of DEGs and enrichment analysis

**Fig. 4:**
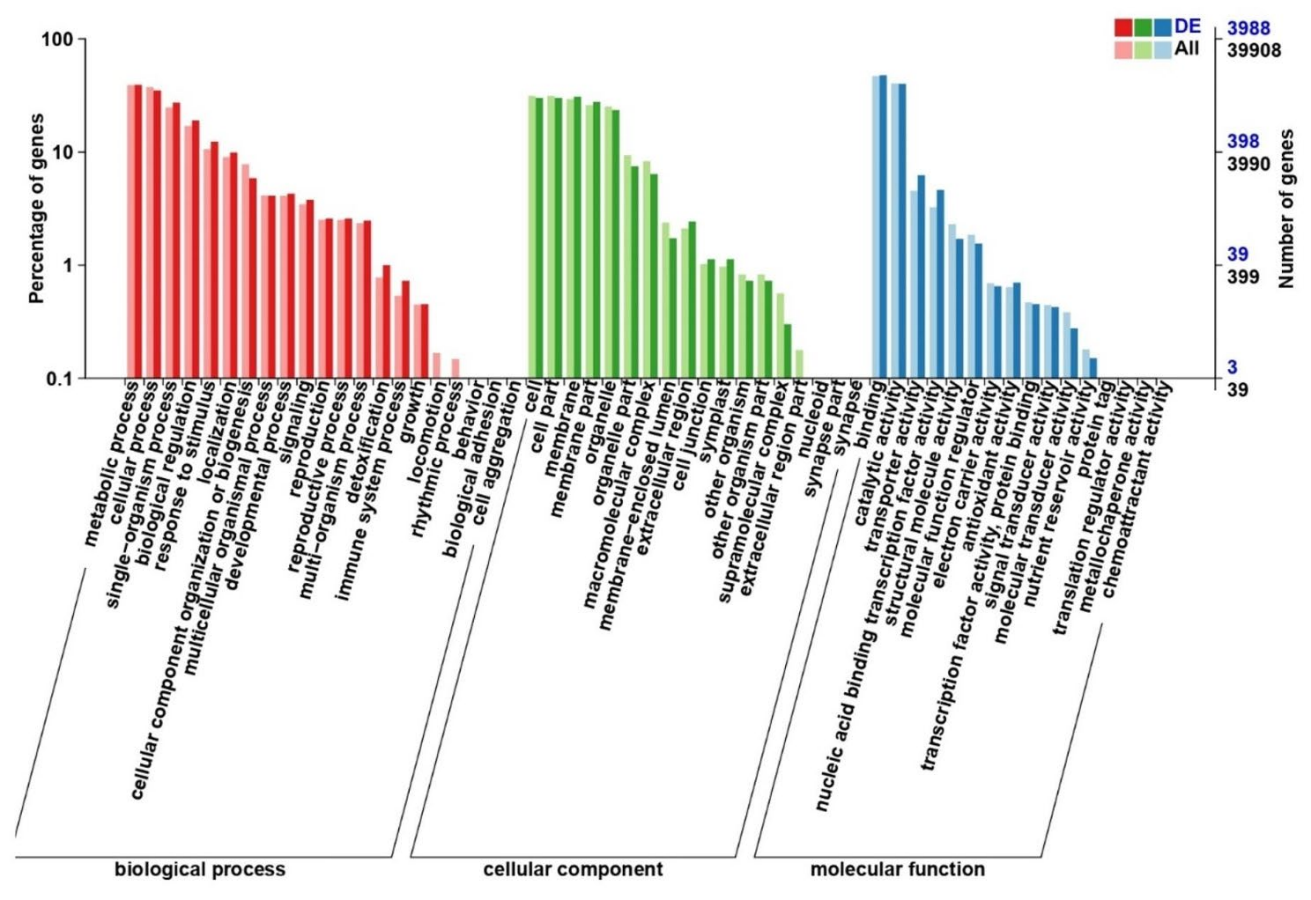
GO classification of DEGs between the two Chinese kale lines

We performed GO, COG, KEGG Orthology (KOG), and KEGG analyses to identify genes involved in the production of anthocyanins in Chinese kale. To predict the molecular function of these genes, we used biological process (BP), cellular component (CC), and molecular function (MF) classifications as the basis for the GO enrichment analysis (Fig. 4, Table S7). We found genes representing a total of 55 enriched terms, and the GO-CC annotation analysis detected 18 enrichments, including extracellular region (GO:0005576), membrane (GO:0016020), nucleoid (GO:0009295), and organelle part (GO:0043226). In GO-MF, we detected 16 enriched terms, including catalytic activity (GO:0003824), binding (GO:0003723), and molecular function regulator (GO:0098772). GO-BP enrichment analysis detected 21 enriched terms, including cellular process (GO:0009987), metabolic process (GO:0008152), response to stimulus (GO:0050896), and single-organism process (GO:0044699).

**Fig. 5:**
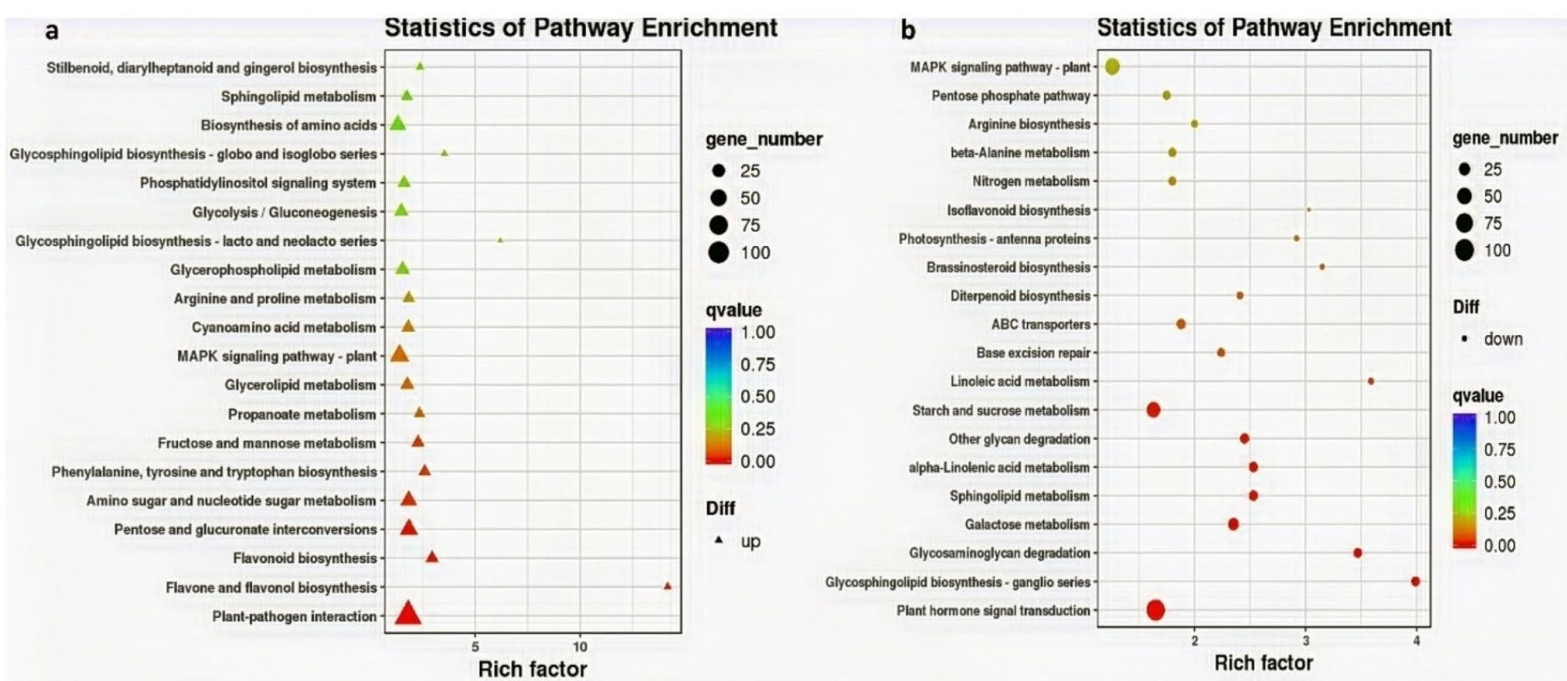
KEGG enrichment pathways of DEGs between purple- and green-stalked Chinese kale. KEGG enrichment pathways of upregulated DEGs (**a**) and downregulated DEGs (**b**)

To further explore their biological roles, we mapped the DEGs to particular KEGG pathways. Specifically, 5,826 DEGs were mapped to 130 KEGG pathways. Significantly enriched pathways were plant-pathogen interaction (Ko04626), amino sugar and nucleotide sugar metabolism (Ko00520), plant hormone signal transduction (Ko04075), MAPK signaling pathway–plant (Ko04016), flavone and flavanol biosynthesis (Ko00944), flavonoid biosynthesis (Ko00941), glycerolipid metabolism (Ko00561), and brassinosteroid biosynthesis (Ko00905) (Fig. 5a, b, and Table S8). Notably, pigment-related pathways were significantly enriched among the DEGs, providing important clues about the processes underlying anthocyanin biosynthesis in Chinese kale.

### Identification of candidate DEGs involved in anthocyanin biosynthesis

**Fig. 6:**
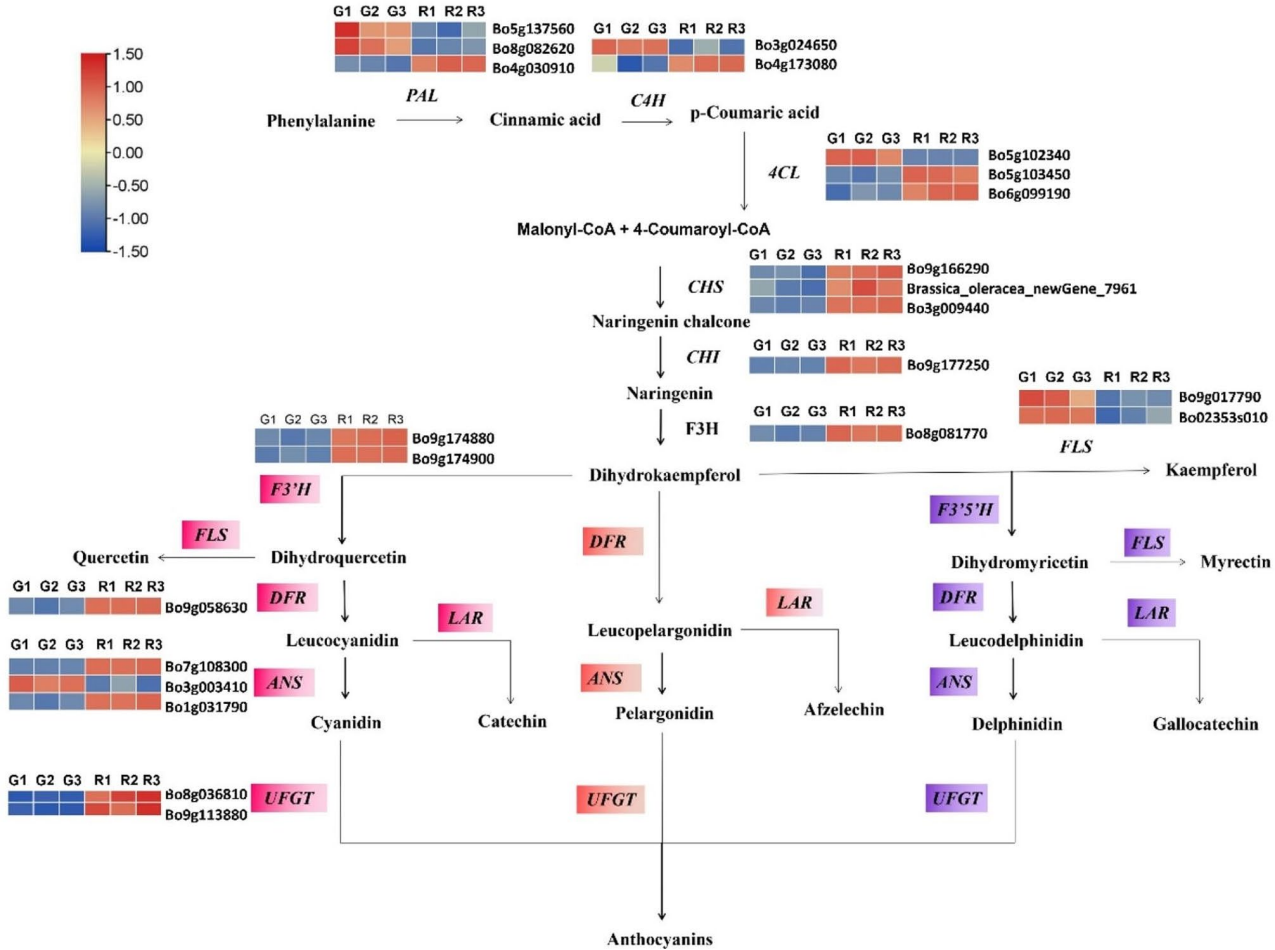
Analysis of the expression of genes involved in anthocyanin biosynthesis in Chinese kale. The illustration shows gene expression levels in the six cDNA libraries created from purple and green-stalked Chinese kale (red = higher expression) in the context of the anthocyanin biosynthesis process using structural genes showing variable expression

Anthocyanin biosynthesis is regulated by a variety of regulatory pathways in plants, with anthocyanin metabolism comprising one of the branches of the flavonoid metabolic pathway. In this study, we found 83 DEGs in this pathway, of which 23 were structural genes related to anthocyanin biosynthesis, including 3 *PAL*, 3 *CHS*, 3 *4CL*, 1 *CHI*, 2 *F3’H*, 1 *F3H*, 2 *C4H*, 2 *FLS*, 3 *ANS*, 1 *DFR*, and 2 *UFGT* (Fig. 6), which is consistent with the high-anthocyanin phenotype of purple-stalked kale. Therefore, it is likely that these particular DEGs contribute to the development of variously colored stalks in Chinese kale.

### Identification of relevant transcription factors

Numerous plant TFs control the production of anthocyanins. MYB TFs, including MYB11, MYB12, MYB113, and others, are part of MYB-bHLH-WD40 (MBW) complex [38]. Proteins in a MBW complex work together in a coordinated manner to activate the appropriate target genes involved in anthocyanin biosynthesis [30, 39]. The MYB TFs within the MBW complex regulate the expression of genes encoding key enzymes of the anthocyanin biosynthesis pathway, such as CHI, CHS, and DFR [40–42]. Activation of these genes leads to the synthesis of anthocyanin pigments and the subsequent development of colorful and nutritious plant tissues.

**Fig. 7:**
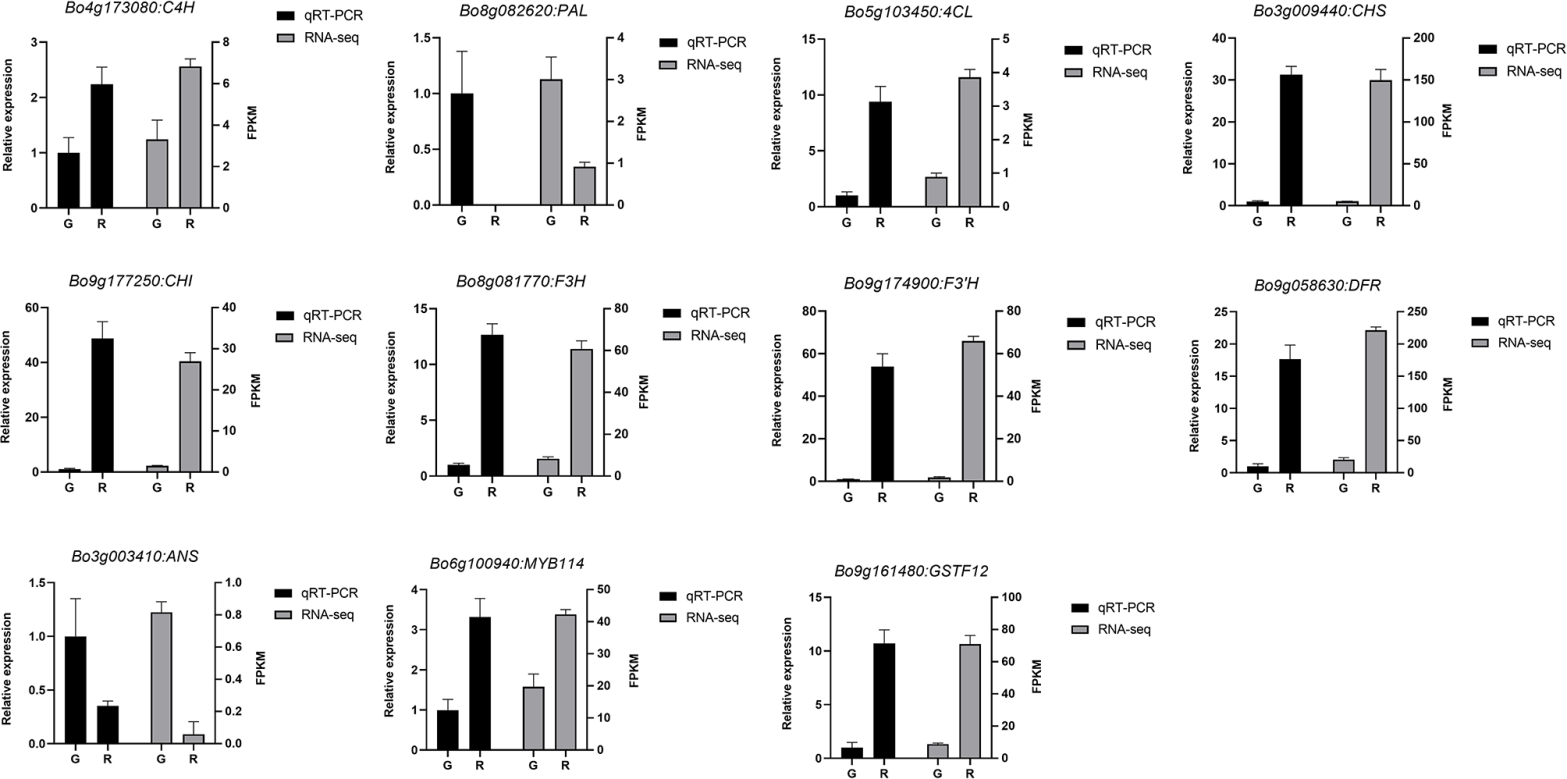
qRT-PCR verification of anthocyanin-related gene expression. Data, from left to right, are represented as relative expression and fragments per kilobase million (FPKM), respectively. Bars show means ± SD of biological replicates data

In this study, we identified a total of 92 anthocyanin-related TF genes, of which 56 were downregulated, and 36 were upregulated (Table S9). Most of these TFs were MYB, bHLH, and WD40 proteins, components of the MBW complex (Table S9). Of the differentially expressed TF genes related to the anthocyanin biosynthesis pathway, *BoMYB114* (Bo6g100940) was shown by qRT-PCR verification to be more highly expressed in purple-stalked Chinese kale vs. the green-stalked variety (Fig. 7). Moreover, supporting our prediction of the importance of *BoMYB114* in anthocyanin production in purple-stalked Chinese kale, we observed a higher expression level of three *CHS* genes, one *CHI* gene, and one *DRF* gene in purple-stalked Chinese kale than in green-stalked Chinese kale.

These findings provide the basis to further analyze the role of *BoMYB114* in controlling anthocyanin biosynthesis and accumulation in *B. oleracea*. Furthermore, we identified BoTT8 (Bo9g086910), a bHLH TF homologous to AtTT8, which was reported to be involved in anthocyanin synthesis in *Arabidopsis* [43]. Transcriptome results showed that *Bo9g086910* gene expression in purple-stalked Chinese kale was higher than in green-stalked Chinese kale (Table S9), suggesting its involvement in anthocyanin biosynthesis. On the other hand, the WD40 TF gene Bo7g096780 was not found to be differentially expressed when comparing purple- and green-stalked Chinese kale.

### Expression profiles of genes in the anthocyanin biosynthetic pathway

In order to verify the accuracy of the transcriptome data, qRT-PCR of 11 anthocyanin synthesis–related genes were performed, and these verification results were consistent with the transcriptome results (Fig. 7). The expression levels of *C4H*, *4CL*, *CHS*, *CHI*, *F3H*, *F3’H*, *DFR*, *MYB114*, and *GSTF12* in purple-stalked Chinese kale were upregulated in RNA-seq as well as in our qRT-PCR analysis, indicating that the transcriptome data were accurate.

### Correlation analysis between selected DEGs and anthocyanins

To identify key candidate genes involved in anthocyanin accumulation in Chinese kale, we conducted correlation analyses between identified 25 DEGs (23 structural genes and 2 *GSTF* genes encoding glutathione *S*-transferases) and 3 metabolites, including cyanidin-3,5-*O*-diglucoside (cyanin), cyanidin-3-*O*-(6″-*O*-p-hydroxybenzoyl) sophoroside-5-*O*-glucoside, and cyanidin-3-*O*-(6″-*O*-feruloyl) sophoroside-5-*O*-glucoside. In all cases, we detected significant correlations (correlation coefficient, R^2^ > 0.8) between our selected DEGs and metabolites (Table S10).

**Fig. 8:**
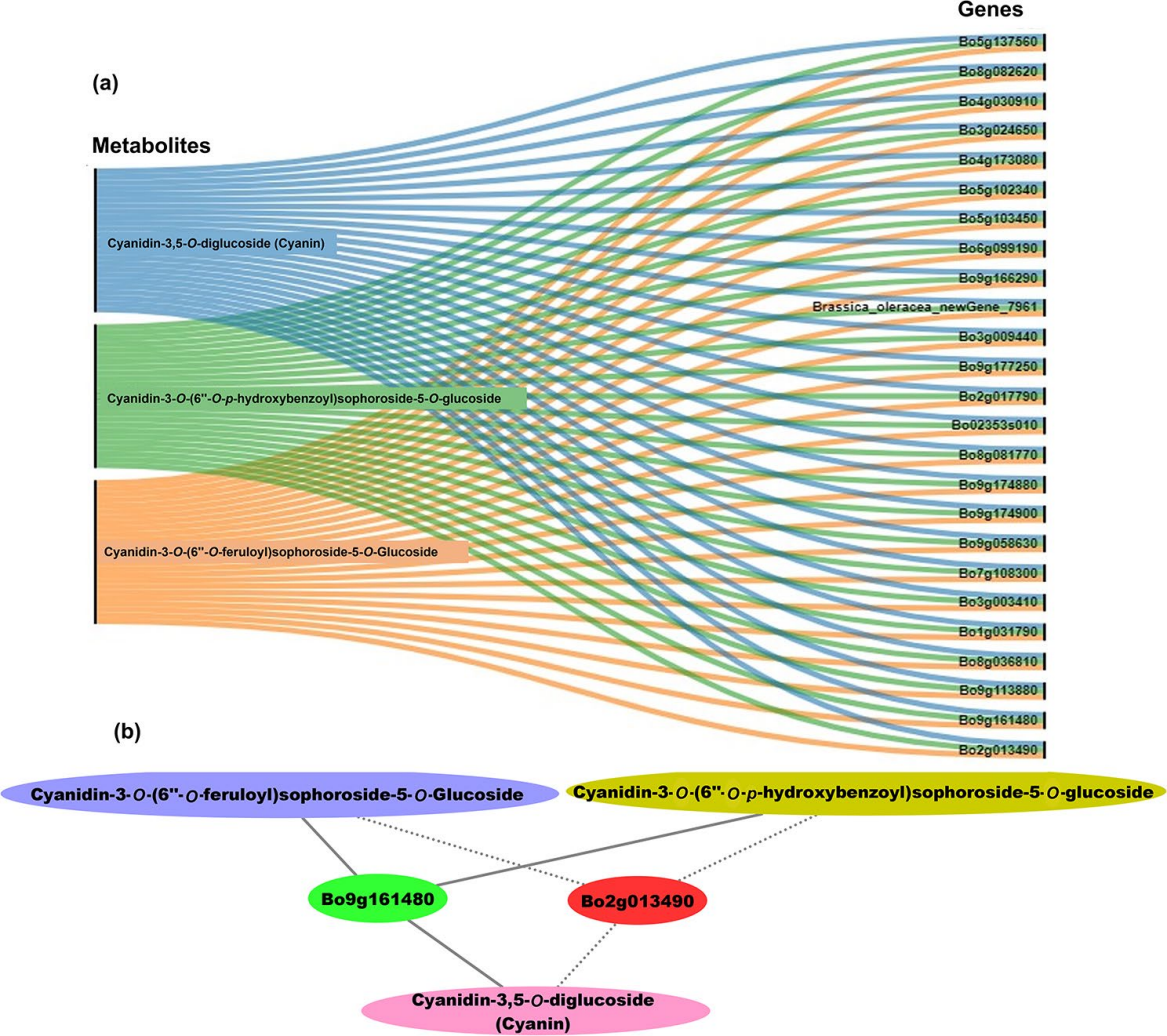
Connection network between core genes and anthocyanin metabolites. (**a**) Network showing relationships between 25 core genes and 3 anthocyanins. (**b**) Network for *GSTF12* (Bo9g161480 and Bo2g013490) genes and three anthocyanins. Solid lines, stronger interaction; dotted lines, weaker interaction

Based on correlation coefficient results, our network analysis showed that all of the selected DEGs were strongly related to these three anthocyanin metabolites (Fig. 8a), demonstrating that these are core genes for anthocyanin accumulation in Chinese kale. Of these, two GSTF subfamily genes *GSTF12* (Bo9g161480 and Bo2g013490) were highly correlated with anthocyanin metabolites (Fig. 8b and Table S10). Specifically, of these two genes, *GSTF12* (Bo9g161480) showed a strong interaction with all three anthocyanins as compared to *GSTF12* (Bo2g013490) (Fig. 8b).

### Identification of *BoGSTF12*

**Fig. 9:**
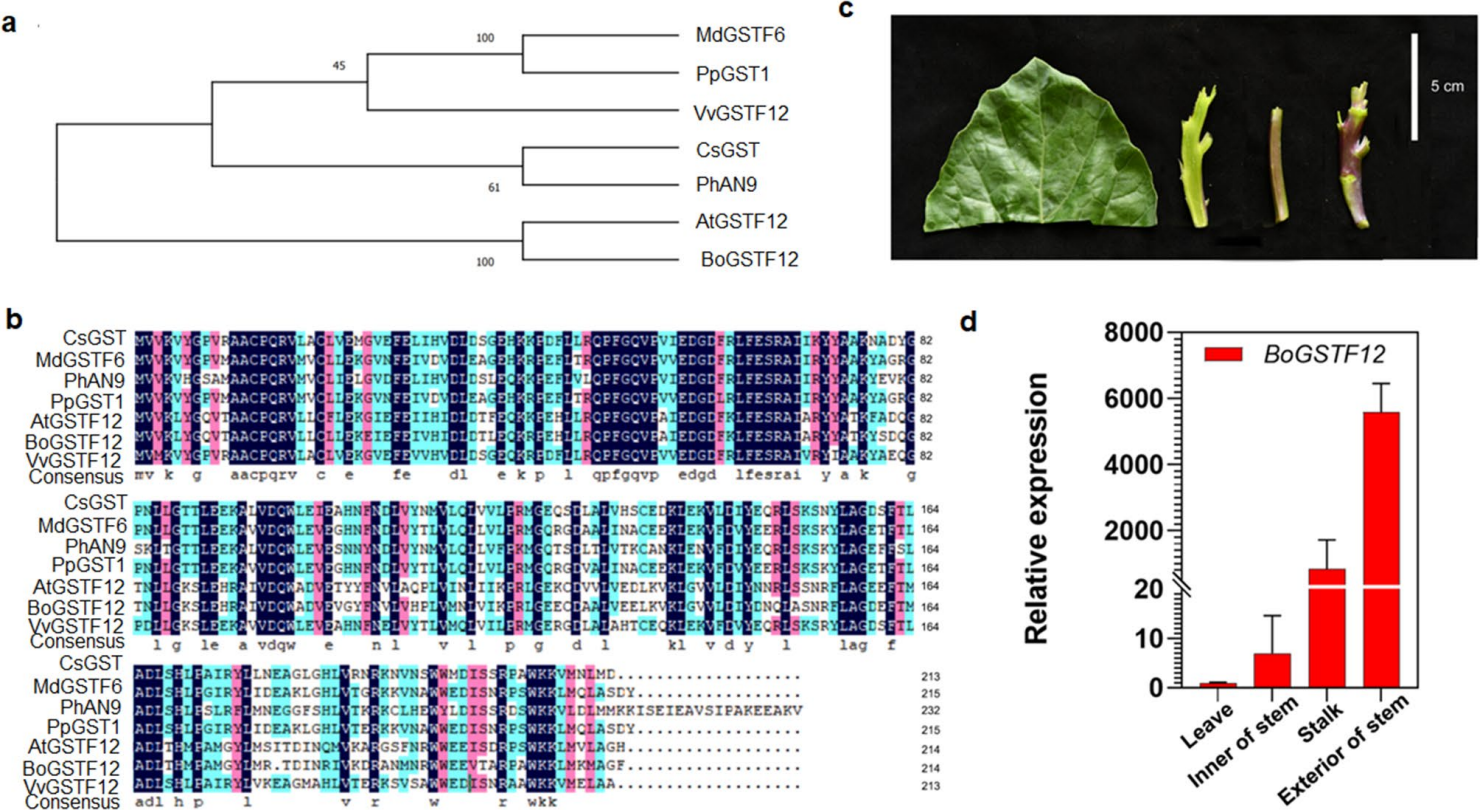
Phylogenetic analysis and expression pattern of *BoGSTF12* (**a**) Phylogenetic analysis, (**b**) multiple sequence alignment of *GST* genes in different species (**c**) phenotypic representation of anthocyanin content in Chinese kale (**d**), Relative expression of *BoGSTF12* in different plant parts of purple-stalked Chinese kale. Scale bar in (**c**) = 5 cm

There are 14 different subgroups of *GST* genes in plants, and the *GSTF* subfamily is a significant class of genes regulating anthocyanin transport. The results of association analysis indicate that *GSTF12* may be involved in the transport of anthocyanins. Therefore, we constructed a phylogenetic tree and sequence alignment between GSTF12 and GSTFs that have been reported to be involved in anthocyanin transport in other species. The result of phylogenetic analysis (Fig. 9a) and sequence alignment (Fig. 9b) showed that *BoGSTF12* was homologous with *AtGSTF12*, which was reported to be involved in anthocyanin transport. Combining the results of transcriptome analysis and qRT-PCR results, we found that the expression level of *BoGSTF12* (Bo9g161480) in purple-stalked Chinese kale was higher than that in green-stalked Chinese kale. Because its expression level was consistent with the trend of anthocyanin content in each tissue of Chinese kale (Fig. 9c-d), we speculate that *BoGSTF12* (Bo9g161480) regulates anthocyanin transport.

### Overexpression of *BoGSTF12* (*Bo9g161480*) gene in *Arabidopsis*

**Fig. 10:**
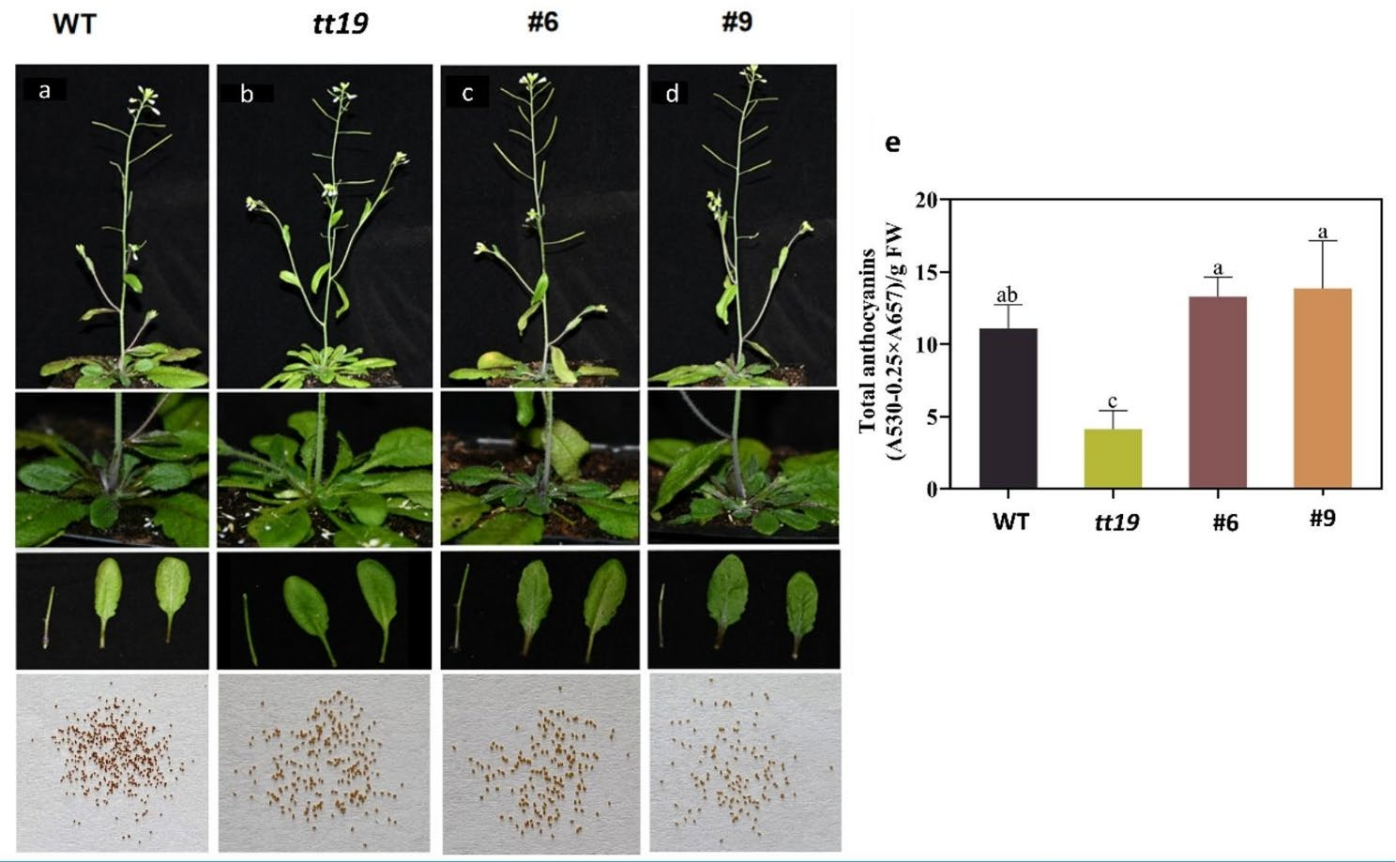
Overexpression of *BoGSTF12* in the *Arabidopsis tt19* mutant and measurements of total anthocyanins contents. (**a**) Phenotypes of wild-type (WT) *Arabidopsis*, (**b**) an *Arabidopsis* knockout mutant of the anthocyanin transporter *AtGSTF12* (*tt19*), and (**c**, **d**) two transgenic lines of *35 S::BoGSTF12-FLAG* in the *tt19* background. (**e**) Total contents of anthocyanins as measured in the infiltration patches; data are means ± SD obtained from three biological replicates. The different letters denote significant differences according to one-way analysis of variance (ANOVA) (*P* < 0.05)

Because *BoGSTF12* is homologous to *Arabidopsis ATGSTF12*, to further explore the function of *BoGSTF12*, it was introduced into the *Arabidopsis tt19* mutant (SALK_105779, a deletion mutant of *Arabidopsis AtGSTF12*). This mutant lacks anthocyanins in the area between the stem and rosette leaves and lacks proanthocyanidins in the seed coat. Two transgenic lines (#6 and #9) were selected for subsequent experiments. *BoGSTF12* rescued the anthocyanin-loss phenotype in the area between the stem and rosette leaves of Arabidopsis *tt19*, but it could not rescue this phenotype in the mutant seeds (Fig. 10a–d). To verify this finding, we measured anthocyanin contents in the *Arabidopsis* WT, *ttl9*, #6, and #9 plants. The stalks of the #6, #9, and WT plants contained more anthocyanins than did *ttl9* plants (Fig. 10e). The above results indicated that *BoGSTF12* was involved in the transport of anthocyanins, but not in the transport of proanthocyanidins, demonstrating a function different from that of *AtGSTF12*.

## Discussion

The goal of the current work was to clarify the molecular processes responsible for the eye-catching purple coloration seen in the stalks of purple-stalked Chinese kale, a popular leafy green vegetable with documented health advantages. *Brassica* vegetables such as broccoli (*Brassica oleracea* var. *italica*), heading Chinese cabbage (*Brassica rapa* ssp. *pekinensis*), mizuna (*Brassica rapa* var. *japonica*), and ornamental cabbage (*Brassica oleracea* var. *acephala*) have attracted a lot of attention because of their high anthocyanin levels [23, 44–46]. Using a thorough integration of metabolomic and transcriptome analyses, we have discovered important metabolites and genes involved in the manufacture of the pigments and other secondary metabolites that are responsible for the distinct purple-stalk phenotype.

### Metabolomic analysis identified anthocyanins causing the purple-stalk phenotype in Chinese kale

Anthocyanins are some of the most important pigments affecting the color of plant tissues. The majority of research on anthocyanins in *Brassica* crops has focused on separating and distinguishing among metabolites [47]. The purple variety of cauliflower (*Brassica oleracea* var. *botrytis*) contains a cyanidin 3-(coumaryl-caffeyl) glucoside-5-(malonyl) pigment [48]. Additionally, using HPLC-ESI-MS/MS, red cabbage (*Brassica oleracea* var. *capitata*) was found to contain over 30 different cyanidin compounds [49].

In this study, our metabolomic analysis revealed a significant accumulation of anthocyanins, flavonoids, and related compounds in purple-stalked Chinese kale compared to the green-stalked variety. These findings are consistent with previous reports that anthocyanins are responsible for the purple coloration in various plant tissues, including leaves, flowers, and stems. Our identification of specific anthocyanin derivatives, such as cyanidin-3-*O*-(6″-*O*-feruloyl) sophoroside-5-*O*-glucoside, cyanidin-3,5-*O*-diglucoside (cyanin), and cyanidin-3-*O*-(6″-*O*-*p*-hydroxybenzoyl) sophoroside-5-*O*-glucoside (Fig. 2a-c), has provided insights into the origin of the diversity of pigments contributing to the purple color. Additionally, we identified other secondary metabolites, such as phenolic acids and flavonols, identified in higher quantities in the purple stalks, suggesting a potential interplay between different classes of metabolites in generating the observed pigmentation.

### Transcriptome analysis revealed candidate genes involved in anthocyanin biosynthesis and transport

One of products of the flavonoid production pathway are anthocyanins, with many structural genes encoding TFs and enzymes that regulate anthocyanin metabolite production. Studies have shown that the majority of the fundamental genes in the anthocyanin production pathway are more highly expressed during vegetative growth in red cabbage than in green cabbage, resulting in a range of leaf colors [50]. Differently colored mizuna, *Arabidopsis*, and other plants have been shown to have coordinated expression of *DFR*, *F3H*, *ANS*, *UFGT*, and *F3’H* [51–53]. Dihydrokaempferol, dihydromyricetin, and dihydroquercetin have a particular substrate bias in DFR derived from different plants [53]. Additionally, ANS, an important enzyme near the end of the anthocyanin synthesis pathway, catalyzes the conversion of monochrome to colored anthocyanins [51]. In our research, comparable outcomes were seen, we found 23 structural genes specifically related to anthocyanin biosynthesis, including three *PAL*, two *C4H*, three *4CL*, three *CHS*, one *CHI*, one *DFR*, three *ANS*, two *FLS*, two *F3’H*, one *F3H*, and two *UFGT* genes (Fig. 6; Table 1). The expression levels of *C4H*, *CHI*, *CHS*, *F3’H*, *4CL*, *F3H*, and *DFR* were significantly higher in the purple-stalked Chinese kale than in the green variety. Our transcriptomic analysis complemented and verified the metabolomic data by providing a broader understanding of the genetic regulation underlying the accumulation of anthocyanin in the purple stalks. The upregulation of these key genes suggests an enhanced flux through the anthocyanin pathway, leading to the increased production of anthocyanin precursors and ultimately the pigments responsible for the purple color.

**Table 1:** DEGs involved in the regulation of anthocyanin levels in purple- and green-stalked Chinese kale

Gene ID	Gene name	Average FPKM of green-stalked Chinese kale	Average FPKM of purple-stalked Chinese kale	Log2-fold change	FDR
Bo5g137560	*PAL*	2.562997333	0.708516667	−1.72	3.52E-09
Bo8g082620	*PAL*	3.009089667	0.916190667	−1.63	3.38E-13
Bo4g030910	*PAL*	2.598262	26.19135667	3.31	1.24E-154
Bo3g024650	*C4H*	2.172948	0.592897	−1.74	3.79E-08
Bo4g173080	*C4H*	3.310021333	6.834617333	1.04	4.83E-08
Bo5g102340	*4CL*	4.910253667	0	−5.63	1.17E-28
Bo5g103450	*4CL*	0.890123333	3.861082667	2.04	2.05E-19
Bo6g099190	*4CL*	0.990403333	4.934258667	2.24	2.79E-25
Bo9g166290	*CHS*	20.92536667	88.70838433	2.09	1.17E-121
Brassica_oleracea_newGene_7961	*CHS*	1.332888667	11.211386	2.97	4.90E-39
Bo3g009440	*CHS*	5.216807667	149.7133487	4.83	0
Bo9g177250	*CH1*	1.513418	26.94277267	4.03	6.17E-96
Bo2g017790	*FLS*	15.01445933	4.946323667	−1.57	1.75E-26
Bo02353s010	*FLS*	3.005581	0.497476333	−2.26	1.62E-08
Bo8g081770	*F3H*	8.391290667	60.74696733	2.85	2.29E-191
Bo9g174880	*F3’H*	2.928916333	75.569687	4.64	0
Bo9g174900	*F3’H*	1.804787333	65.95989	5.05	2.55E-172
Bo9g058630	*DFR*	20.42142	221.340393	3.44	0
Bo7g108300	*ANS*	2.223137	71.22292833	4.95	0
Bo3g003410	*ANS*	0.815995	0.058852667	−2.64	1.17E-06
Bo1g031790	*ANS*	24.01311467	222.6269683	3.21	0
Bo8g036810	*UFGT*	11.39123633	71.48828367	2.65	7.81E-204
Bo9g113880	*UFGT*	2.362485	17.00569033	2.82	2.35E-83

Anthocyanins are very prone to degradation and instability. Glucosyltransferase (GST), which determines the position of glycosylation, is crucial for the stability and solubility of plant flower color and anthocyanins. As a result, anthocyanins can function as pigments in vacuoles in a similar manner to flavonoid 3-*O*-glucosyltransferase and anthocyanidin 3-*O*-glucosyltransferase [54, 55]. Delphinidin conversion to delphinidin-3-O-glucoside in *Clitoria ternatea* was also reported to be catalyzed by anthocyanidin 3-O-glucosyltransferase (*UGT78K6*) [56]. Additionally, *Freesia hybrida Fh3GT1*, which encodes *UF3GT*, is essential for the production of anthocyanin glycosides [53]. Similar results were found in our study as well (Fig. 6).

TFs are crucial for regulating the enzymes in the anthocyanin production pathway that are involved in the formation of vegetable, fruit, and flower color [49, 57, 58]. Previous research suggested that, in the flavonoid biosynthesis pathway, the late biosynthetic genes (LBGs) were controlled mostly by the transcriptional complexes made of WD-repeat/MYB/bHLH proteins [59, 60]. Increased expression of early biosynthetic genes (EBGs) and LBGs in *Arabidopsis* leaves overexpressing *PAP1* suggest that the phenylpropanoid pathway is responsible for the increased flavonoid pigment content observed [61]. This shows that LBGs may not be the only genes in the flavonoid pathway that are regulated by transcriptional complexes in *Arabidopsis*. Similarly, the coordinated expression of *TRANSPARENT TESTA8* (*TT8*) and *MYB2*-enhanced anthocyanin synthesis may occur by positively activating EBGs and LBGs such as *F3H* and *CHS* in red cabbage and *F3’H ANS*, *DFR*, *LDOX*, *UFGT*, and *GST* in purple-headed Chinese cabbage [50, 62]. Additionally, the expression of the structural genes *F3’H*, *DFR*, *LDOX*, *UGT75C1*, and *GST12* in *Arabidopsis* was impacted by the interaction of *MYB113* or *MYB114* with TTG1 and bHLHs (GL3, EGL3, and TT8) [59]. In our study, we identified the TF *BoMYB114*, of the MYB family, which is known regulator of anthocyanin biosynthesis. The upregulation of these transcription factors suggests their involvement in orchestrating the transcriptional response leading to purple color development in purple-stalked Chinese kale. Additionally, we noted the increased expression level of three *CHS* genes, one *CHI* gene, and one *DRF* gene in our purple-stalked Chinese kale as compared to green-stalked kale, supporting our results that *BoMYB114* is involved in anthocyanin synthesis in purple-stalked Chinese kale. These results provide the basis for further exploration of the functional analysis of *BoMYB114* and its role in regulating anthocyanin production and accumulation in *B. oleracea*.

### GST is a pivotal player in anthocyanin transport

Anthocyanins are produced in the cytosol and accumulate in the vacuole. The processes underpinning the intracellular transport of anthocyanins have been partially elucidated in recent years. GST involvement, membrane transport, and/or vesicle trafficking are required for the transfer of anthocyanins from cytosolic production to vacuolar accumulation [63]. The role of GSTs in anthocyanin transport and accumulation has been confirmed in maize [64], petunia [65], Arabidopsis [66], cyclamen [67], perilla [68], grape [69], apple [70], and litchi [71]. In our study, *BoGSTF12* expression was highly correlated with anthocyanin content, and further functional analysis revealed that *BoGSTF12* rescued the anthocyanin-loss phenotype in *Arabidopsis tt19*, an anthocyanin transport mutant, in the stem and rosette leaves, but not in the mutant seeds (Fig. 10). Similar to our findings, previously identified genes such as *PpGST1* from peach (*Prunus persica L. (Batsch)*) [72], *An9* (*Petunia hybrida*), *LcGST4* (*Litchi chinensis*), strawberry *RAP* (*Fragaria ananassa*), *CsGSTF1* (*Camellia sinensis*), and apple *MdGSTF6* (*Malus domestica*) [70, 71, 73–75], could functionally complement the anthocyanin-less phenotype of *Arabidopsis tt19* mutant, but not the proanthocyanidin-deficient phenotype in the seed coat.

### Implications and future directions

The insights gained from this study have implications for both agricultural and nutritional research. The identification of key genes and metabolites involved in pigment accumulation can inform breeding strategies aimed at enhancing the purple coloration in Chinese kale or related crops. Additionally, the health-promoting properties of the secondary metabolites identified warrant further investigation to confirm their bioactivity and potential contributions to human health. Our integrated metabolomic and transcriptomic analysis provides valuable insights into the molecular basis of the purple-colored stalks in Chinese kale. These findings shed light on the regulatory network governing anthocyanin pigment biosynthesis and lay the groundwork for future studies exploring the functional significance of key metabolites in this nutritionally important vegetable.

## Conclusion

The discovery of the function of *BoGSTF12* in anthocyanin transport and accumulation using a combination of RNA-seq and metabolomic research marks a substantial advancement in our understanding of the molecular processes driving anthocyanin production in plants. This research provides useful knowledge for both horticultural and agricultural applications by dissecting the complex mechanism of anthocyanin production and transport, enabling the development of crops with improved nutritional value and attractive traits. This work also highlights the ability of contemporary -omics technology to decipher the intricacies of plant biology and has the potential to inspire further advancements in farming and plant breeding.

### Electronic supplementary material

Below is the link to the electronic supplementary material.

**Supplementary Material 1: Table S1:** List of primers

**Supplementary Material 2: Table S2:** Sequences used for phylogenetic tree analysis

**Supplementary Material 3: Table S3:** Metabolites identifies in both Chinese kale (GREEN and RED)

**Supplementary Material 4: Table S4:** Sequencing data statistics

**Supplementary Material 5: Table S5:** Mapping rate of sequencing data

**Supplementary Material 6: Table S6:** List of DEGs in Reg and green Chinese kale

**Supplementary Material 7: Table S7:** List of all significant GO enrichment analyses

**Supplementary Material 8: Table S8:** List of all KEGG enrichment pathways

**Supplementary Material 9: Table S9:** List of anthocyanin related TFs

**Supplementary Material 10: Table S10:** Correlation analysis between DEGs and metabolites

## Data Availability

All data generated or analyzed during this study are included in supplementary information.
